# Evaluating machine learning-enabled and multimodal data-driven exercise prescriptions for mental health: a randomized controlled trial protocol

**DOI:** 10.3389/fpsyt.2024.1352420

**Published:** 2024-01-15

**Authors:** Miaoqing Tan, Yanning Xiao, Fengshi Jing, Yewei Xie, Sanmei Lu, Mingqiang Xiang, Hao Ren

**Affiliations:** ^1^Guangzhou Sport University, Guangzhou, China; ^2^China Swimming College, Beijing Sport University, Beijing, China; ^3^China’s National Artistic Swimming Team, Beijing, China; ^4^Institute of Physical Education, Sichuan University, Chengdu, China; ^5^Faculty of Data Science, City University of Macau, Taipa, Macao SAR, China; ^6^Project-China, School of Medicine, The University of North Carolina, Chapel Hill, NC, United States; ^7^College of Business, City University of Hong Kong, Hong Kong, Hong Kong SAR, China; ^8^Programme in Health Services and Systems Research, Duke-NUS Medical School, Singapore, Singapore; ^9^South China Agricultural University, Guangzhou, China; ^10^Institute for Healthcare Artificial Intelligence Application, Guangdong Second Provincial General Hospital, Guangzhou, China

**Keywords:** mental health, exercise prescription, artificial intelligence, personalized medicine, randomized controlled trial

## Abstract

**Background:**

Mental illnesses represent a significant global health challenge, affecting millions with far-reaching social and economic impacts. Traditional exercise prescriptions for mental health often adopt a one-size-fits-all approach, which overlooks individual variations in mental and physical health. Recent advancements in artificial intelligence (AI) offer an opportunity to tailor these interventions more effectively.

**Objective:**

This study aims to develop and evaluate a multimodal data-driven AI system for personalized exercise prescriptions, targeting individuals with mental illnesses. By leveraging AI, the study seeks to overcome the limitations of conventional exercise regimens and improve adherence and mental health outcomes.

**Methods:**

The study is conducted in two phases. Initially, 1,000 participants will be recruited for AI model training and testing, with 800 forming the training set, augmented by 9,200 simulated samples generated by ChatGPT, and 200 as the testing set. Data annotation will be performed by experienced physicians from the Department of Mental Health at Guangdong Second Provincial General Hospital. Subsequently, a randomized controlled trial (RCT) with 40 participants will be conducted to compare the AI-driven exercise prescriptions against standard care. Assessments will be scheduled at 6, 12, and 18 months to evaluate cognitive, physical, and psychological outcomes.

**Expected outcomes:**

The AI-driven system is expected to demonstrate greater effectiveness in improving mental health outcomes compared to standard exercise prescriptions. Personalized exercise regimens, informed by comprehensive data analysis, are anticipated to enhance participant adherence and overall mental well-being. These outcomes could signify a paradigm shift in exercise prescription for mental health, paving the way for more personalized and effective treatment modalities.

**Registration and ethical approval:**

This is approved by Human Experimental Ethics Inspection of Guangzhou Sport University, and the registration is under review by ChiCTR.

## 1 Introduction

Mental illnesses, a significant global health concern, encompass a wide spectrum of psychological disorders affecting millions worldwide (1, 2). The ramifications of these disorders extend beyond the individual, affecting families, communities, and economies. For instance, depression alone is a leading cause of disability worldwide, and mental disorders are among the major contributors to the overall global burden of disease (3, 4). The societal impact of mental illness is profound, encompassing economic costs due to lost productivity, healthcare expenses, and the intangible yet substantial cost of reduced quality of life (5, 6). Additionally, mental health disorders can exacerbate social issues such as homelessness and unemployment, creating a vicious cycle of poverty and illness (7).

The field of exercise prescription for mental illness has garnered increasing attention in recent years, as evidenced by a growing body of research. Studies have consistently demonstrated the efficacy of regular physical activity in alleviating symptoms of various mental disorders, including depression, anxiety, and schizophrenia (8–11). For example, a meta-analysis by Rosenbaum et al. indicated that moderate to vigorous physical activity could significantly reduce depressive symptoms in adults (12). Despite these advancements, several challenges and gaps remain in the application of exercise prescription for mental health. A primary concern is the lack of individualization in exercise regimes. Most existing studies and protocols adopt a one-size-fits-all approach, neglecting the unique needs, preferences, and limitations of individual patients (13). This generalized approach may lead to suboptimal outcomes and lower adherence rates, as patients may find the prescribed exercises either too challenging or not engaging enough. Another significant limitation is the scarcity of data-driven methods in tailoring exercise prescriptions. While some studies have begun to explore the use of data in optimizing exercise interventions (14, 15), there is still a considerable gap in integrating comprehensive, multimodal data – such as genetic, physiological, and behavioral information – to inform exercise prescriptions. This lack of integration results in missed opportunities to enhance the precision and effectiveness of exercise as a therapeutic tool for mental health.

The integration of Artificial Intelligence (AI) in healthcare and medicine has marked a transformative era, particularly with the advancements in deep learning algorithms and the enhanced capabilities in processing large volumes of data (16, 17). These technological leaps have enabled the deployment of AI across various medical fields, including mental health, internal medicine, infectious disease control, heart failure management, and diabetes care, among others. Specifically in the domain of mental health (18), AI-driven tools are being used to predict patient outcomes, personalize treatment plans, and even assist in early diagnosis through pattern recognition in patient data. In internal medicine (19), AI algorithms contribute to diagnostic accuracy and patient management, while in the field of infectious diseases control (20), AI plays a pivotal role in outbreak prediction, tracking, and formulating response strategies. The application of AI is equally significant in managing chronic diseases. For instance, in heart failure (21, 22), AI assists in patient monitoring, risk assessment, and tailoring treatment regimes. Similarly, in diabetes management (23), AI technologies are employed for continuous glucose monitoring and predicting episodes of hypoglycemia or hyperglycemia, thereby enhancing patient care. In the context of China’s medical resource constraints, the development of AI-based prescription recommendation systems is particularly promising (24). These systems have the potential to mitigate the gap in healthcare delivery, offering efficient, accurate, and accessible medical advice. A notable example of such innovation is the reinforcement learning-based dynamic prescription recommendation system proposed by Wang and colleagues (25). This system exemplifies the application of AI in optimizing treatment plans, adapting to patient-specific needs and changes over time (26).

In the burgeoning field of exercise prescription, the application of AI and machine learning has begun to show promising results. Tuka and Linhart have explored the potential of these technologies for creating personalized exercise prescriptions, tailoring recommendations to the unique needs and conditions of patients (27). Additionally, this research underscores the superiority of current AI technology over traditional methods in exercise prescription. AI systems offer a higher degree of personalization and adaptability, leveraging real-time data to tailor exercise plans more effectively to individual needs and conditions. This approach signifies a significant shift from traditional, generalized exercise guidelines to more individualized, data-driven strategies.

In a more targeted study, Chen and colleagues have devised a hierarchical learning framework specifically designed for crafting physical exercise prescriptions for Chinese children (28). This innovative framework takes into account various factors such as age, physical development, and individual health conditions, demonstrating the effectiveness of AI in addressing the diverse needs of specific populations. However, despite these advancements, there remains a notable gap in the literature regarding the application of machine learning in the context of exercise prescription for mental health. Mental illness presents unique challenges and necessitates tailored approaches in exercise prescription, considering factors like psychological state, medication side effects, and the fluctuating nature of symptoms. To bridge this gap, our research aims to develop an interpretable, machine learning-based intelligent system dedicated to exercise prescription for the prevention and management of mental illness. This system will not only adapt to the individual needs of patients but also provide insights into the rationale behind each prescription, ensuring transparency and trust in AI-driven recommendations.

## 2 Methods

### 2.1 Study design

In the first phase, we focus on developing a multimodal data-driven intelligent system for exercise prescription. To achieve this, we will initially recruit 1,000 participants for AI model training and testing. Of these, 80% (800 participants) form the training set. To enhance the robustness and generalizability of the AI model, we will use ChatGPT to simulate an additional 9,200 cases based on the initial 800, resulting in a comprehensive training set of 10,000 instances. The remaining 20% (200 participants) will be reserved as a testing set. This diverse dataset will then be annotated by a team of 10 experienced doctors from the Department of Mental Health at Guangdong Second Provincial General Hospital. Their expertise will ensures the accuracy and clinical relevance of the data labeling, which is critical for the development of a reliable and effective AI system.

In our analysis, we will set the effect size at 0.5 to assess the intervention’s effectiveness. This value represents a medium effect size, which is considered substantial in the context of our study. Following the development and preliminary testing of the AI model, the second phase of the study will involves an RCT with 50 participants. These participants are randomly divided into two groups: 25 in the experimental group and 25 in the control group. The experimental group will receives personalized exercise prescriptions generated by the newly developed AI system, while the control group will receives standard exercise recommendations. This phase aims to evaluate the efficacy of the AI-driven exercise prescription system in a real-world clinical setting. The RCT’s design ensures that the results are robust and can be attributed specifically to the intervention, thereby providing strong evidence of the system’s potential benefits for individuals with mental illnesses.

**Figure 1:**
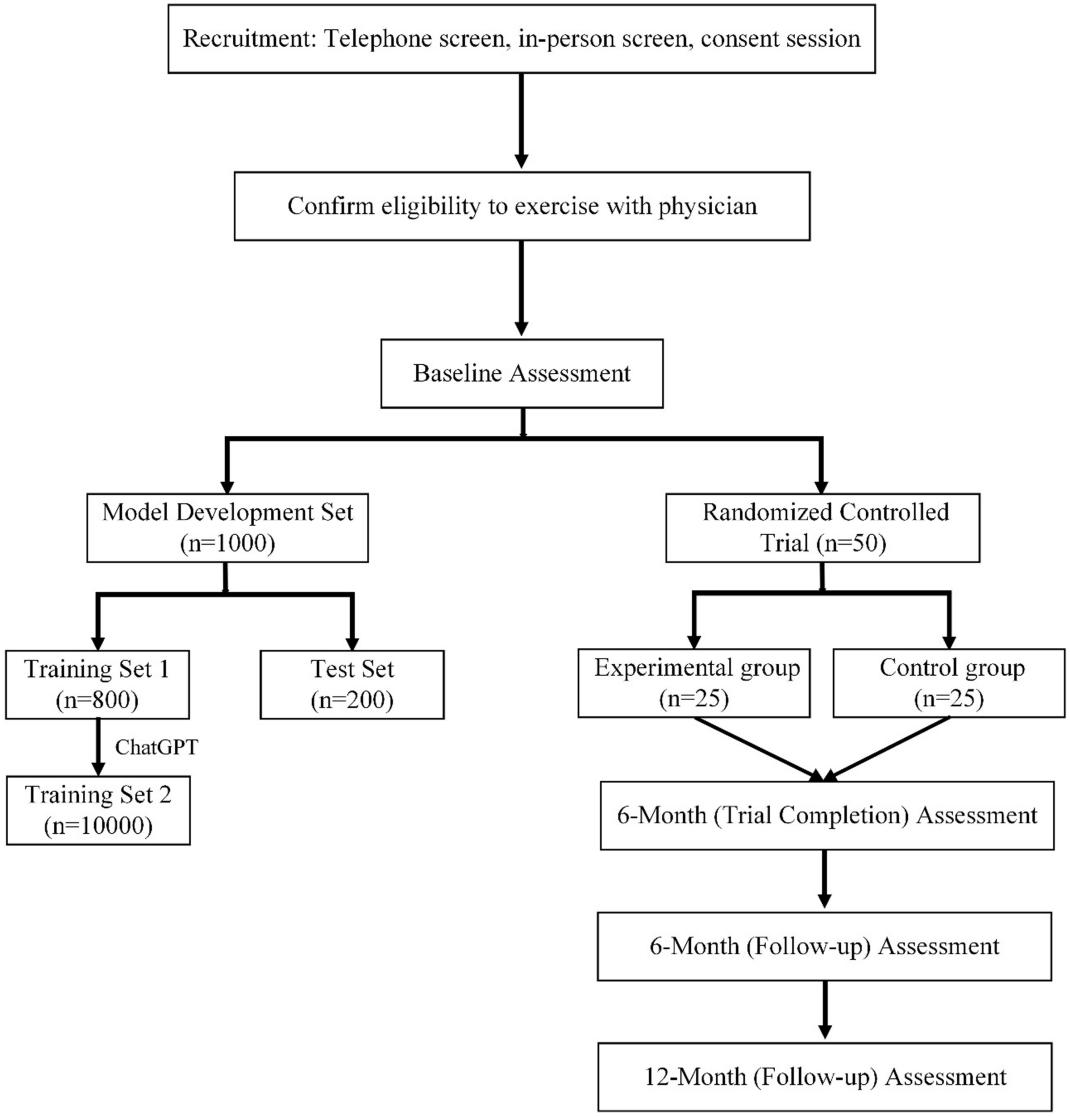
Overview of study design incorporating AI model development and randomized controlled trial phases.

The effectiveness of the AI-driven exercise prescriptions is evaluated at multiple time points: initially at the end of the 6 months trial period (Trial Completion Assessment), followed by a 6 months follow-up assessment and a 12 months follow-up assessment. These assessments are designed to track and compare the short-term and long-term impacts of the AI-driven exercise prescriptions on the mental health outcomes of the participants (shown in Figure 1).

### 2.2 Recruitment

Participants for the study will be recruited from the general community and the Department of Mental Health at Guangdong Second Provincial General Hospital, utilizing platforms like WeChat or other social media to effectively reach and engage potential participants. To reach a diverse pool of potential participants, advertisements will be placed in community centers and local newspapers in Guangzhou. At the Department of Mental Health, individuals receiving care will have the opportunity to consent to the use of their medical records for research purposes and express their interest in participating in research studies. Prospective participants will initially undergo a telephone screening process to determine their eligibility. This screening will include an evaluation based on predefined inclusion and exclusion criteria, alongside the Physical Activity Readiness Questionnaire for Everyone (PAR-Q Plus) (29), a widely recognized tool for assessing an individual’s readiness for physical exercise. Upon passing the initial screening, eligible participants will be invited to a detailed consent and screening session. This session, which can be conducted either over the phone or in person, will provide comprehensive information about the study, including its objectives, methodology, potential risks, and benefits. During this session, research staff will ensure that participants fully understand the study and are comfortable with their involvement, thereby upholding the highest standards of ethical research practice. For participants involved in the AI part of data collection, we are offering a subsidy of 100 RMB per person. This incentive is designed to encourage their active participation and consistent engagement with the data collection process. Additionally, for participants in the RCT, we are providing a more substantial subsidy of 300 RMB per person.

### 2.3 Time frame

The enrollment of participants for this study will commenced on January 1, 2024. This will mark the beginning of a crucial phase in our research, where we started gathering data from a diverse group of individuals, crucial for the development and testing of our AI model. The final assessment of the study is anticipated to be completed by January 1, 2027. This assessment will mark the conclusion of the two-year follow-up period post the trial completion.

### 2.4 Inclusion criteria

In this study, participants are selected based on specific inclusion criteria to ensure both relevance and safety. Individuals aged 18 to 65, diagnosed with a mental illness such as depression, anxiety disorders, bipolar disorder, or schizophrenia, are eligible. They must be in a stable medical condition, with no acute mental health crises in the past 6 months, and have the cognitive ability to understand and consent to the study procedures. Physical capability for exercise, assessed by the PAR-Q Plus, is required, alongside a willingness to adhere to the study’s protocol. Participants should not be engaged in any other structured exercise program, have access to communication tools for remote monitoring, and reside within a distance from the study site in the Guangzhou area. These criteria are designed to ensure that participants are representative of the target population and can safely and effectively engage in the study.

### 2.5 Exclusion criteria

Certain exclusion criteria are established to maintain the integrity and safety of the research. Individuals are excluded if they are under 18 or over 65 years of age, to focus the study on a specific adult demographic. Those without a clinically confirmed diagnosis of mental illness, or those experiencing acute mental health crises or hospitalizations within the last 6 months, are also excluded. Participants with severe cognitive impairments that prevent understanding of the study or informed consent, as well as those with physical conditions or disabilities that contraindicate exercise, based on the PAR-Q Plus assessment, are not eligible. Additionally, individuals already engaged in a structured exercise program or unable to commit to the study’s schedule and protocols are excluded. Further, lack of access to necessary communication tools for remote monitoring or residing outside the practical geographical scope of the study (beyond the Guangzhou area) also leads to exclusion. These criteria ensure that participants are well-suited to the study’s aims and methods while safeguarding their well-being.

### 2.6 Data entry

Throughout the data collection process, no personal identifiers will be gathered to ensure participant confidentiality. All data collected on paper will be securely stored in locked cabinets, accessible only to authorized study personnel. Trained staff will be responsible for entering alphanumeric data, implementing range checks to verify the accuracy of data values. Both alphanumeric data and neuroimaging files will be stored on a secure server managed by UBC, adhering to strict data protection protocols. To further safeguard participant privacy, all collected data will undergo a de-identification process, removing any potential links to individual identities.

## 3 Machine learning model development

### 3.1 Model inputs

In our study, a range of comprehensive assessment tools have been meticulously chosen to serve as inputs for the machine learning model (shown in Table 1). These include the State and Trait Anxiety Inventory (STAI), Center for Epidemiologic Studies Depression Scale (CES-D), Pittsburgh Sleep Quality Index (PSQI), and several others, each uniquely contributing to a multi-dimensional understanding of participant well-being. The collected data from these tools encompass various aspects of mental health, physical activity, social engagement, and general well-being. For instance, the STAI provides insights into anxiety levels, the CES-D focuses on symptoms of depression, while the PSQI assesses sleep quality. Other questionnaires like the CHAMPS Physical Activity Questionnaire and the Lubben Social Network Scale offer valuable data on physical activity habits and social network strengths, respectively. This diverse dataset is fed into the machine learning model to analyze patterns, correlations, and potential predictors of mental health outcomes.

**Table 1:** Comprehensive assessment tools utilized as input for machine learning model.

Questionnaires	Description
Mindfulness attention awareness scale (MAAS) (36)	This 12-item questionnaire focuses on assessing mindfulness during daily activities like conversation, commuting, and eating, underlining the importance of mental presence in everyday life
CHAMPS physical activity questionnaire for older adults (37)	A specialized 41-item questionnaire that gauges the weekly frequency and duration of physical activities particularly relevant to older adults
Sedentary behavior questionnaire (SBQ) (38)	This questionnaire specifically measures the time spent in nine different sedentary behaviors during a typical weekday and weekend, highlighting patterns of physical inactivity
Lubben social network scale – revised (LSNS-R) (39)	A 12-item questionnaire evaluating social engagement with family and friends, crucial for understanding social support networks
Social provisions scale (SPS) (40)	A 24-item scale that measures the availability and adequacy of social support in an individual’s life
UCLA Loneliness scale (41)	A 20-item scale focused on assessing feelings of loneliness and social isolation, reflecting on the emotional aspects of social health
Health care resource utilization (HRU) (42)	A 10-item questionnaire that asks participants about their health care visits, services used, and their ability to perform chores, aimed at calculating the economic burden of health care needs
State and trait anxiety inventory (STAI) (43)	This comprehensive 40-item questionnaire evaluates mood and levels of anxiety, providing valuable insights into the psychological well-being of participants
Center for epidemiologic studies depression scale (44)	A detailed 20-item questionnaire designed to measure symptoms associated with depression, focusing on experiences over the past week
Pittsburgh sleep quality index (PSQI) (45)	An in-depth 19-item questionnaire that assesses sleep quality over the previous month. It employs subjective ratings across seven different components, including sleep quality, latency, duration, habitual efficiency, disturbances, use of sleeping medication, and daytime dysfunction
STOP bang questionnaire (46)	An 8-item tool aimed at assessing the risk of obstructive sleep apnea, crucial for understanding sleep-related health risks
Life space questionnaire (47)	A 6-item questionnaire that measures the mobility extent of older adults, providing insights into their functional range and independence
Everyday memory questionnaire (EMQ) (48)	A comprehensive 28-item questionnaire that evaluates memory failures occurring in the past 3 months, offering a window into cognitive health

### 3.2 Data annotation

In the crucial phase of data annotation, our study collaborates with the Department of Mental Health at Guangdong Second Provincial General Hospital, enlisting the expertise of five experienced physicians. The data annotation process involved five doctors, each with over 10 years of experience. The dataset was divided equally among them, with each doctor responsible for annotating a specific portion. This approach ensured that the entire dataset was annotated efficiently and effectively, with all doctors completing their assigned tasks until the full dataset was annotated.

The physicians, utilizing their medical expertise and understanding of mental health, carefully review the responses from the various questionnaires, including the STAI, CES-D, and others. The doctors consider several critical aspects of exercise prescription: (1) Type of Exercise: determining the most suitable form of physical activity, whether it’s aerobic, strength training, flexibility exercises, or a combination, based on the participant’s health status and preferences; (2) Frequency: establishing how often the participant should engage in the prescribed exercises, balancing effectiveness with practicality. (3) Intensity: setting the appropriate level of exertion for each exercise, tailored to the individual’s physical capabilities and mental health needs. (4) Duration: recommending the length of each exercise session to maximize benefits while ensuring safety and adherence.

The initial dataset comprised 800 cases, designated as the training set, and a smaller set of 200 cases, set aside as the test dataset. To augment the robustness and diversity of our training dataset, we employed the *ChatGPT* to generate an additional 9,200 simulated samples. This step was based on the patterns and characteristics observed in the initial 800 cases. By doing so, we significantly expanded our dataset, enriching the training process and enhancing the model’s ability to generalize across a broader range of scenarios. This extensive data annotation and augmentation process is crucial for developing an accurate and effective AI-driven exercise prescription system. It ensures that the model is not only trained on a substantial and varied dataset but also fine-tuned to reflect real-world complexities and nuances in mental health and physical fitness.

### 3.3 Model selection

In the model selection phase of our study, we employed a multi-model approach to identify the most effective machine learning algorithm for our data. We will rigorously trained and tested various models, including Linear Regression, Decision Trees, Random Forests, Support Vector Machines (SVMs), Neural Networks, Recurrent Neural Network (RNN), and Gradient Boosting Machines (GBMs), each evaluated on accuracy, precision, recall, and F1 score. The process involved using our annotated dataset for training and a separate testing set for validation. Ultimately, the model demonstrating the highest predictive accuracy and robustness on the testing set will be selected. This model, excelling in processing complex mental health data, forms the cornerstone of our AI-driven exercise prescription system, ensuring personalized and effective recommendations for individuals with mental health conditions. In our study, we anticipate that the AI model will achieve an AUC of over 0.9, demonstrating its high accuracy in tailoring exercise prescriptions for mental health improvements.

### 3.4 Experiment setup

Our predictive models were constructed using Python 3.7.13, leveraging libraries such as Pandas for data manipulation, scikit-learn for machine learning algorithms, and NumPy for numerical computations.

## 4 Randomized controlled trial

### 4.1 RCT design and methods

Our study employs a parallel-group RCT to evaluate the effectiveness of an AI-based exercise prescription system in mental health management. Participants are divided into two groups: an intervention group and a control group, each consisting of 25 individuals. This balanced design allows for a robust comparison of outcomes between the groups, thereby providing reliable evidence on the impact of the AI-driven intervention. The randomization process involves a computer-generated sequence, ensuring an unbiased assignment of participants into the intervention group (*n* = 25) and the control group (*n* = 25). Stratification is conducted based on key variables such as age, gender, and specific mental health diagnoses to maintain comparable groups. This methodology is critical for mitigating potential confounding factors and ensuring the validity of the study results. In the intervention group, each participant receives a personalized exercise prescription tailored by the AI system, based on their unique health profile. This approach aims to maximize the efficacy of the exercise regimen in improving mental health outcomes. The control group, on the other hand, receives standard care practices, which may include general health advice or standard exercise recommendations. The distinction in treatment between the groups is central to evaluating the added value of the AI-driven exercise prescriptions.

### 4.2 Intervention measures

The 25 participants in the intervention group receive personalized exercise prescriptions generated by our AI model. These prescriptions are meticulously tailored based on each participant’s health profile and mental health status. The AI model determines the most suitable Type of Exercise for each individual, ranging from aerobic activities to strength training, depending on their physical and mental health needs. The Frequency of exercise is set, aiming for a balance that maximizes benefit while considering each individual’s lifestyle and capacity. Intensity levels are also customized, ensuring that exercises are challenging yet safe and achievable for each participant. Lastly, the Duration of each exercise session is specified by the AI model, optimizing the time spent on each activity for maximum efficacy. For example, for a participant with moderate depression and a sedentary lifestyle, the AI model might prescribe light aerobic activities like brisk walking or cycling for 30 min, three times a week. The intensity would be set at a moderate level, ensuring the participant can comfortably sustain the activity while gaining mental health benefits. The model may also suggest gentle yoga twice a week to improve flexibility and reduce stress, tailoring the duration to 20 min per session to match the participant’s initial physical fitness level. The intervention lasts for a period of 4 weeks, during which participants adhere to their personalized exercise regimen. The control group, comprising another set of 25 participants, receives standard care practices. This generally includes generic health advice and non-tailored exercise recommendations, reflecting the conventional approach to mental health management. The control group’s treatment does not involve the AI-driven customization of exercise parameters, serving as a baseline to evaluate the effectiveness of the personalized exercise prescriptions provided to the intervention group.

### 4.3 Outcome assessment and follow-up

This study employs a rigorous and comprehensive assessment schedule, as outlined in Table 2, to evaluate a broad spectrum of outcomes across different time intervals: at 6 months (Trial Completion), 12 months (First Follow-up), and 18 months (Second Follow-up) (detail shown in Table 2).

**Table 2:** Schedule of assessments and follow-up intervals for study period.

Assessments	Study period		
	Trial Completion (6 months)	Follow-up (12 months)	Follow-up (18 months)
MoCA (30) and MMSE (31)	✓		✓
IADL (32)	✓		
Subjective memory complaints (33)		✓	
Descriptors	✓		✓
Rey-0 complex figure, Judgement of line orientation, prospective memory, spatial memory, Kirby delay discounting	✓		
Physical performance	✓		
Short physical performance battery	✓		✓
400 m walk test	✓		
30 s sit-to-stand	✓		✓
Reproductive, Florida cognitive activitylifetime stimulation, and epic-Norfolk physical activity questionnaire (34)	✓		
FCl, PSOl, and life space questionnaire (35)	✓	✓	✓
State and trait anxiety inventory	✓		✓
Health related quality of life and sedentary behavior	✓	✓	✓

**Cognitive assessments**: the MoCA (Montreal Cognitive Assessment) and MMSE (Mini-Mental State Examination) are conducted at the trial completion (6 months) and again at the 18 months follow-up. These tools are essential for measuring cognitive function and detecting any changes over time.

**Functional and physical assessments**: the study includes the Instrumental Activities of Daily Living (IADL) assessment at the 6 months mark to evaluate functional abilities. Physical performance is measured at 6 months using tests such as the Short Physical Performance Battery, the 400 m Walk Test, and the 30 s sit-to-stand test. These assessments are repeated at the 18 months follow-up to track physical endurance and mobility changes.

**Memory and psychological assessments**: subjective memory complaints are evaluated at the 12 months follow-up, while other detailed memory tests like the Rey-O Complex Figure and Judgement of Line Orientation are conducted at 6 months. The State and Trait Anxiety Inventory is administered at both the 6 months and 18 months follow-ups to assess changes in anxiety levels.

**Lifestyle and quality of life questionnaires**: the study also incorporates the Reproductive, Florida Cognitive Activity Lifetime Stimulation, and Epic-Norfolk Physical Activity Questionnaire at the 6 months mark. In addition, the FCI (Frailty Criteria Index), PSQI (Pittsburgh Sleep Quality Index), and Life Space Questionnaire are administered at all three assessment points (6, 12, and 18 months) to monitor lifestyle changes and quality of life.

**Health-related assessments**: health-related quality of life and sedentary behavior are evaluated at all three time points to provide a comprehensive view of the participants’ overall health status and lifestyle habits.

### 4.4 Ethics consideration

The study protocol was approved by Guangzhou Sport University (ID number: 2023LcLL-71).

### 4.5 Statistical analysis

In the initial phase of our statistical analysis, we will employ descriptive statistics to summarize the participant characteristics, including demographics, baseline health measures, and other relevant variables. This step will involve calculating means, standard deviations, and proportions to provide a clear overview of the study population. Following this, comparative analyses between the intervention and control groups will be conducted using independent *t*-tests (or Mann–Whitney U tests for non-normally distributed data) for continuous variables, and Chi-square tests (or Fisher’s exact tests) for categorical variables. This will enable us to detect any significant differences arising from the intervention.

Considering the multiple assessment points in our study, longitudinal data analysis techniques will be crucial. These methods, including repeated measures ANOVA or mixed-model ANOVAs, will allow us to track and analyze changes over time both within and between participant groups. This approach is essential for understanding the dynamics of the intervention’s impact, accounting for both individual variations and time-dependent factors.

To quantify the intervention’s effectiveness, we will calculate effect sizes, providing a measure of the practical significance of our findings. The handling of missing data, a common challenge in longitudinal studies, will be addressed through methods like multiple imputation or last observation carried forward, ensuring the robustness of our results. All statistical tests will be two-tailed, with a significance level set at *p* < 0.05. We plan to use sophisticated statistical software such as SPSS or R for these analyses, enabling us to apply the most appropriate and advanced statistical techniques for our data.

## 5 Discussion

This study represents a significant step forward in the field of exercise prescription for mental health, leveraging the power of AI and machine learning to tailor interventions to individual needs. Our findings contribute to a growing body of evidence underscoring the importance of personalized healthcare approaches, particularly in managing mental illnesses.

The application of a multimodal data-driven AI system in developing exercise prescriptions has demonstrated significant promise in enhancing mental health outcomes. This innovative approach effectively counters the limitations inherent in traditional exercise regimes, which often adopt a generalized approach, potentially overlooking individual nuances. Our AI-driven system, by incorporating a diverse array of data points including mental health status, physical capabilities, and personal lifestyle preferences, offers interventions that are far more customized and aligned with each participant’s unique needs.

This level of individualization in exercise prescription is pivotal (49). It recognizes the complex interplay between mental health and physical activity, allowing for adjustments based on factors such as an individual’s specific mental health condition, their physical fitness levels, and their daily routines and preferences. For instance, someone with mild depression might benefit from a different type and intensity of exercise compared to someone with severe anxiety. Likewise, a physically active individual might be prescribed a more vigorous regimen than someone who is less active. By tailoring exercise recommendations in this way, our AI system not only caters to the distinct needs of each participant but also maximizes the potential for adherence. Adherence is often a significant challenge in traditional exercise regimes, but by offering personalized and therefore more relevant and engaging exercise plans, our system could significantly improve compliance rates (50).

Moreover, the enhanced efficacy of these personalized exercise regimes could lead to better mental health outcomes. Regular, tailored physical activity can positively impact various aspects of mental health, including mood elevation, reduction in anxiety symptoms, and overall improvement in mental well-being. This suggests that personalized exercise prescriptions, as adjuncts to conventional mental health treatments like psychotherapy and medication, could offer a comprehensive approach to mental health care.

While our research has yielded encouraging outcomes, it is not without limitations. The complexity of mental health disorders and the variability in individual responses to exercise highlight the challenges in developing universally effective AI-driven interventions. Additionally, the reliance on self-reported data in some of our assessments could introduce bias or inaccuracies. Future research should aim to incorporate more objective measures and explore the long-term sustainability of AI-prescribed exercise regimes.

## 6 Conclusion

In conclusion, this study marks a significant stride in the realm of mental health care, demonstrating the potential of a multimodal data-driven AI system to revolutionize exercise prescription for individuals with mental illnesses. While acknowledging the complexities inherent in mental health disorders and the limitations of our current approach, our findings underscore the promise of personalized, AI-enabled exercise regimens in enhancing mental well-being. The integration of sophisticated AI with individualized exercise prescriptions paves the way for more effective, patient-tailored therapeutic strategies, offering a glimpse into the future of mental health treatment. Moving forward, it is imperative to address the identified limitations through more extensive research, aiming to refine the AI algorithms further and explore the long-term impact of these personalized interventions, ultimately contributing to more comprehensive and effective mental health care.

## Ethics statement

The studies involving humans were approved by the Human Experimental Ethics Inspection of Guangzhou Sport University. The studies were conducted in accordance with the local legislation and institutional requirements. The participants provided their written informed consent to participate in this study.

## Author contributions

MT: Conceptualization, Funding acquisition, Investigation, Writing – original draft. YaX: Formal analysis, Investigation, Writing – original draft. FJ: Conceptualization, Project administration, Resources, Software, Supervision, Writing – review & editing. YeX: Data curation, Methodology, Validation, Writing – review & editing. SL: Resources, Visualization, Writing – review & editing. MX: Funding acquisition, Project administration, Supervision, Writing – review & editing. HR: Conceptualization, Investigation, Methodology, Supervision, Visualization, Writing – original draft, Software.
